# Controllable Synthesis and Tunable Photocatalytic Properties of Ti^3+^-doped TiO_2_

**DOI:** 10.1038/srep10714

**Published:** 2015-06-05

**Authors:** Ren Ren, Zhenhai Wen, Shumao Cui, Yang Hou, Xiaoru Guo, Junhong Chen

**Affiliations:** 1 Department of Mechanical Engineering, University of Wisconsin-Milwaukee, 3200 North Cramer Street, Milwaukee, WI 53211, USA

## Abstract

Photocatalysts show great potential in environmental remediation and water splitting using either artificial or natural light. Titanium dioxide (TiO_2_)-based photocatalysts are studied most frequently because they are stable, non-toxic, readily available, and highly efficient. However, the relatively wide band gap of TiO_2_ significantly limits its use under visible light or solar light. We herein report a facile route for controllable synthesis of Ti^3+^-doped TiO_2_ with tunable photocatalytic properties using a hydrothermal method with varying amounts of reductant, i.e., sodium borohydride (NaBH_4_). The resulting TiO_2_ showed color changes from light yellow, light grey, to dark grey with the increasing amount of NaBH_4_. The present method can controllably and effectively reduce Ti^4+^ on the surface of TiO_2_ and induce partial transformation of anatase TiO_2_ to rutile TiO_2_, with the evolution of nanoparticles into hierarchical structures attributable to a high pressure and strong alkali environment in the synthesis atmosphere; in this way, the photocatalytic activity of Ti^3+^-doped TiO_2_ under visible-light can be tuned. The as-developed strategy may open up a new avenue for designing and functionalizing TiO_2_ materials for enhancing visible light absorption, narrowing band gap, and improving photocatalytic activity.

Since Fujishima discovered the photocatalytic splitting of water by using titanium dioxide (TiO_2_) electrodes, TiO_2_ has become the most attractive photocatalyst because of its multiple advantages, such as structural stability, abundance, environmentally-friendliness, and low-cost^1^,^2^. However, the relatively wide band gap in TiO_2_ greatly hinders efficiently harvesting solar energy for applications in photocatalysis, solar cells, and photoelectrochemical cells. Accordingly, significant research has been devoted to understanding the fundamental processes and exploring routes to enhance the photocatalytic activity and efficiency of TiO_2_^3^,^4^. Fortunately, continuous breakthroughs have been made in the preparation, functionalization, and modification of TiO_2_-based photocatalysts to improve the absorption of visible light (~50% of solar light) for photocatalytic applications^5^,^6^,^7^. In general, modifying TiO_2_ with a suitable dopant not only changes the mechanism and kinetics under UV irradiation but also introduces more visible-light activity that is absent with pure TiO_2_^8^. Three strategies have been proposed to advance properties and corresponding photocatalytic applications of TiO_2_: 1) impurity-doping or dye-anchoring on TiO_2_ catalysts, which can extend its absorption range to visible light region^9^,^10^,^11^; 2) synthesizing TiO_2_ nanocrystals with specific crystal surface orientations because some specific crystalline planes, e.g., (001) plane, tend to show a higher catalytic activity than others and mixed crystallographic facets^12^,^13^; 3) Fabricating TiO_2_-based nanohybrids with other functional materials, such as carbon nanotubes (CNTs) and graphene, to attain a synergistic effect between them^14^,^15^,^16^.

Recently, Chen *et al.* reported a conceptually different method to improve solar absorption ability by introducing disorders in the surface layers of nanophase TiO_2_, i.e., Ti^3+^-doped TiO_2_^17^. The study showed that disorder-engineered TiO_2_ nanocrystals exhibit substantially improved solar-driven photocatalytic activities for photo-oxidation of organic molecules and water splitting. Unfortunately, the preparation processes had to be conducted in a high-pressure hydrogen system for a reaction period of as long as five days, which leads to disadvantages of long reaction time, low yield, and more waste residues. Therefore, it is highly desirable to develop improved methods for fabrication of such Ti^3+^-doped TiO_2_. Many investigations have demonstrated that Ti^3+^-containing (blue) TiO_2_ that contains oxygen vacancies exhibit significant photocatalytic activity in the visible light region; however, the catalyst could not maintain such activity for a sufficiently long period of time^18^. In addition, hierarchically structured TiO_2_-based materials were reported to improve the performance of the materials because their highly porous structures were beneficial for enhancing the utilization efficiency of light^19^,^20^. However, the capability of visible light absorption still needs further improvement. TiO_2_-based photocatalysts synthesized by hydrothermal treatment have drawn great attention since hydrothermal methods possess advantages of convenience, relatively low processing temperature, and high yield^21^,^22^,^23^. Although NaBH_4_ was previously reported for reducing TiO_2_ through a hydrothermal method^24^,^25^,^26^,^27^,^28^,^29^, the resulting photocatalytic performance was inadequate because only a small amount of NaBH_4_ was used. Fang *et al.* added amount of NaBH_4_ during the synthesize process but no more than 0.4 g, which is may insufficient to enable the formation of defective or partially reduced TiO_2_^30^. In summary, there is still lack of comprehensive and systematic investigation and discussion to study how NaBH_4_ affect the morphology, structure, and photocatalytic activity of the reducing TiO_2_.

In the present research, a systematic research was reported by preparing of a series of Ti^3+^-doped TiO_2_ by tuning the amount of NaBH_4_, yielding color changes of the TiO_2_ products from white, light yellow, light grey, to dark grey with the increasing amount of NaBH_4_. More importantly, we firstly reported an increased concentration of NaBH_4_ applied in the hydrothermal reaction would facilitate the conversion of anatase TiO_2_ into rutile TiO_2_ with the evolution of nanoparticles into hierarchical structures thanks to a high pressure and strong alkali environment in this system. Moreover, it is demonstrated that the as-developed Ti^3+^-doped TiO_2_ with a mixed phase and nanostructure can potentially lower the recombination rate of electron-hole pairs due to the presence of Ti^3+^ and oxygen vacancies that are able to trap photo-excited electrons on the surface.

## Results

**Figure 1:**
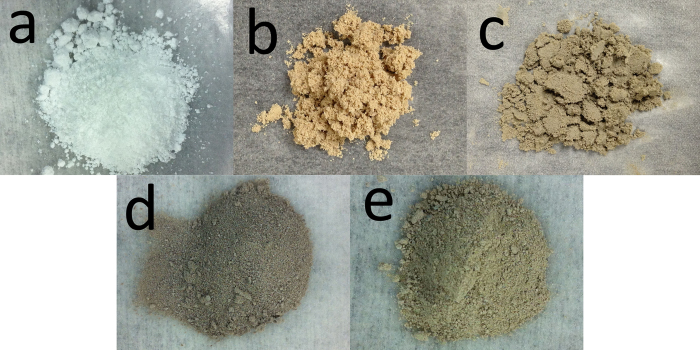
Photographs of pristine TiO_2_ (**a**) and doped TiO_2_ samples, (**b**) TiO_2_-1, (**c**) TiO_2_-2, (**d**) TiO_2_-3 and (**e**) TiO_2_-4.

Reduced TiO_2_ samples were synthesized by adding different amounts of sodium borohydride (NaBH_4_) in the hydrothermal reaction at 180 ∞C for 16 hours. Specifically, 0, 2, 7, 10 and 12 g NaBH_4_ were used in separate experiments; and the as-obtained products were denoted as pristine TiO_2_, TiO_2_-1, TiO_2_-2, TiO_2_-3 and TiO_2_-4, respectively. Figure 1 shows the digital photographs of the series of TiO_2_ samples. With the increasing amount of NaBH_4_, the color of the resulting powders changes from light yellow for TiO_2_-1, light grey for TiO_2_-2, dark grey for TiO_2_-3, to light grey for TiO_2_-4, and all of these samples show a striking contrast to the white color of the pristine TiO_2_. These results indicate that the hydrothermal treatment, which occurs at a mild reaction temperature, high-pressure, and a reduced atmosphere, did affect the surface properties of TiO_2_.

**Figure 2:**
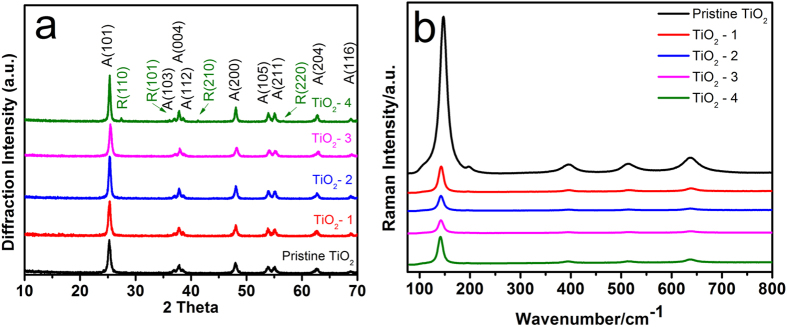
(**a**) Representative XRD pattern and (**b**) Raman spectra of the pristine TiO_2_ and as-synthesized TiO_2_.

To determine the crystal structure and possible phase changes during the hydrothermal synthesis, X-ray diffraction (XRD) was carried out to study the series of samples during the evolution process (Fig. 2a). All of the samples show diffraction peaks matching well with the crystal structure of the anatase phase TiO_2_ (71–1169, JCPDS). No new XRD peaks are observed for samples with 2, 7, and 10 g of NaBH_4_, i.e., TiO_2_–1, TiO_2_–2, and TiO_2_–3. However, a set of diffraction peaks appear at 27.4°, 36.1°, 44.1° and 56.6° for TiO_2_-4; these four peaks can be well indexed to the characteristic peaks of (110), (101), (210), and (220) crystal planes of rutile phase TiO_2_ (75–1751, JCPDS), suggesting that TiO_2_–4 contained both anatase phase and rutile phase TiO_2_. The average crystallite size of TiO_2_ was estimated according to the Scherrer’s equation (1)


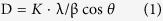


where K is the Scherrer constant, λ, the X-ray wavelength, β, the peak width of half maximum, and θ is the Bragg diffraction angle. The particle sizes for pristine TiO_2_, TiO_2_-1, TiO_2_-2, TiO_2_-3 and TiO_2_-4 are 15.20 nm, 16.36 nm, 16.55 nm, 16.84 nm, and 19.76 nm, respectively. The intensities of the diffraction peaks became weaker with the increase of the amount of NaBH_4_ from 2 g to 10 g, suggesting a decreased crystallinity for TiO_2_ samples after the hydrothermal treatment possibly due to the formation of defects under a relative higher pressure in a reducing environment. The crystalline degree in turn grew stronger with further increasing the amount of NaBH_4_ to 14 g, which can be attributed to the increased pressure promoting the reorganization or restructuring of crystallites, thereby leading to the enhancement of the product crystallinity^31^,^32^. Raman spectroscopy was also used to characterize the series of TiO_2_ samples (Fig. 2b). Raman peaks appear at 147, 397, 515, and 637 cm^-1^ corresponding to E_g_, B_1g_, A_1g_, and E_g_ lattice vibration modes, respectively, which indicates that all samples are majorly dominated by anatase type titanium dioxide. The Raman bands shift toward a lower wavenumber possibly due to the increase in particle size from pristine sample to reduced sample^33^,^34^.

**Figure 3:**
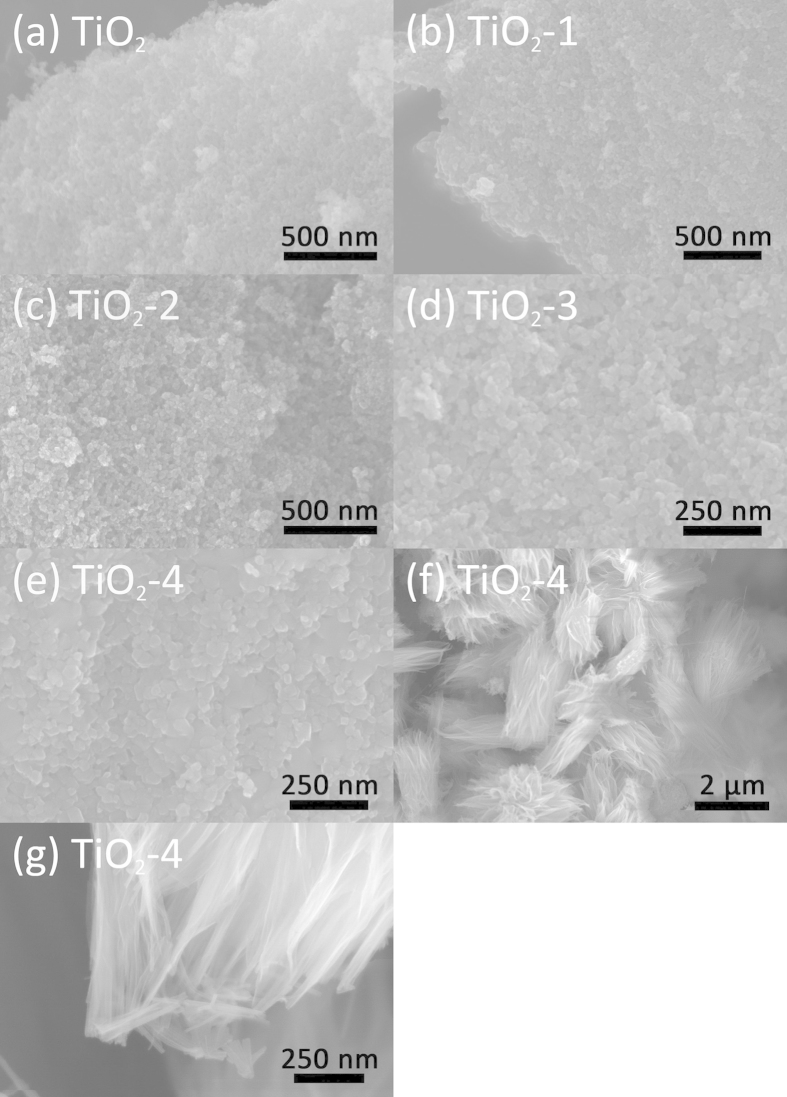
SEM images of pristine TiO_2_ (**a**) and as-obtained TiO_2_ nanostructures: (**b**) TiO_2_-1, (**c**) TiO_2_-2, (**d**) TiO_2_-3 and (**e**) TiO_2_-4; (**f**) and (**g**) hierarchical structures TiO_2_-4.

The morphology and structure of the as-prepared TiO_2_ were further characterized by scanning electron microscopy (SEM). Figure 3a–e present typical SEM images of the pristine TiO_2_, TiO_2_-1, TiO_2_-2, TiO_2_-3, and TiO_2_-4, respectively. The size of TiO_2_ particles increased with increasing the amount of NaBH_4_ in the synthesis process, which is most likely due to the agglomeration of nanoparticles induced by a higher concentration of NaBH_4_. The results are basically in agreement with the particle size calculation by using Scherrer’s equation from XRD results. It should be noted that, for TiO_2_-4, there also appeared some hierarchical microstructures with an average size of 2 to 4 μm that were constructed by a large number of nanofibers about 20-30 nm in diameter, as shown in Fig. 3f,g (ESI, Figs. S1c and S1d). Actually, a small fraction of hierarchical microstructures were also found in the sample TiO_2_-3 (ESI, Fig. S1a and S1b), suggesting gradual evolution of nanostructures from nanoparticles to nanofiber upon tuning the amount of NaBH_4_. Hierarchical structures were previously proven to be beneficial for improving photocatalytic activity because of their special hierarchical porous structure, good permeability, and a large surface area compared with other low dimensional structures^29^,^35^,^36^. Furthermore, the TiO_2_ hierarchical structure can absorb more light through multiple reflections and lead to more photogenerated electrons to participate in the photocatalytic degradation process^37^,^38^. Therefore, TiO_2_-4 is expected to offer enhanced light-harvesting capability and a higher specific surface area than other TiO_2_ samples.

**Figure 4:**
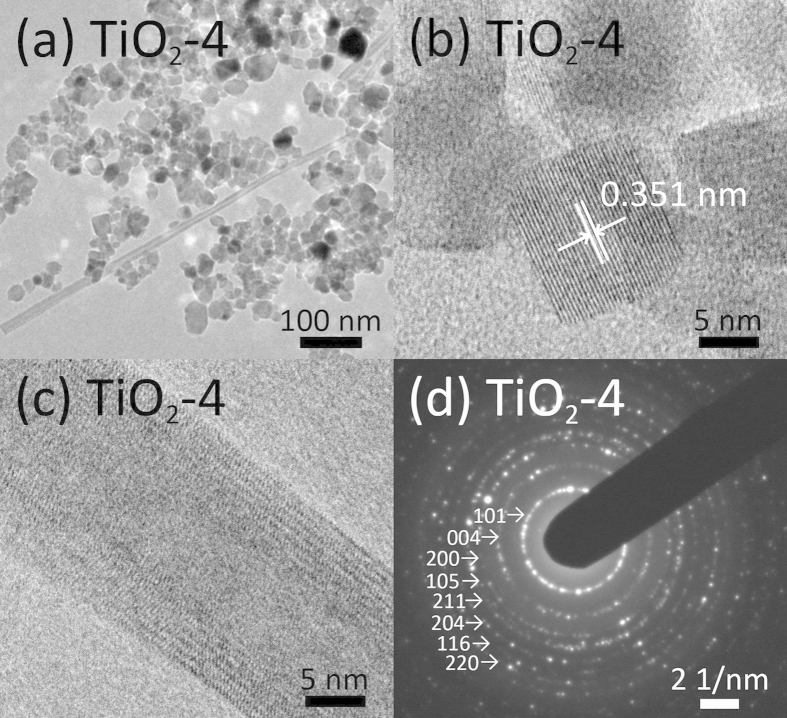
TEM micrographs of sample TiO_2_-4: (**a**) overview image of TiO_2_-4, HRTEM images of TiO_2_-4 nanoparticles (**b**) and nanotube (**c**).(**d**) SAED pattern of synthesized TiO_2_-4.

The morphology and structure of as-prepared TiO_2_ were further elucidated by transmission electron microscopy (TEM) and high-resolution TEM (HRTEM) images shown in Fig. 4. Figure 4a shows the TEM image of nanoparticles from sample TiO_2_-4 with discernible TiO_2_ nanofibers, which is in agreement with the SEM observation. The TiO_2_ nanofibers were formed possibly due to the high pressure during the phase transition process^39^,^40^. Figure 4b,c display the HRTEM images of an individual particle and nanofiber from sample TiO_2_-4, respectively. In addition, a set of well-defined diffraction rings are observed in selected area electron diffraction (SAED) patterns (Fig. 4d), which is in good agreement with the anatase phase of synthesized TiO_2_ nanocrystals^41^. Pristine TiO_2_ nanocrystals show a lattice spacing= 0.350 nm that is close to that of anatase TiO_2_ (101) (0.351 nm). After the hydrothermal treatment by adding different amounts of NaBH_4_, the characteristic TiO_2_-3 (ESI, Fig. S2) and TiO_2_-4 nanocrystal lattice spacing of 0.351 nm corresponds to the (101) lattice plane of anatase TiO_2_, which is consistent with previous results^42^. There is no noticeable change in the nanocrystal lattice spacing value corresponding to the anatase (101) plane, which indicates that the Ti^3+^ has been introduced into the lattice without modifying the dimension of the unit cell^43^.

**Figure 5:**
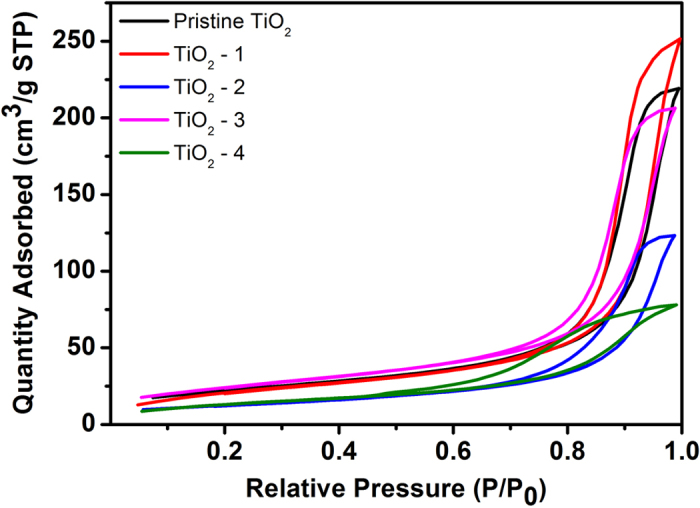
N2 adsorption-desorption isotherms for pristine TiO_2_ and as-obtained TiO_2_.

Figure 5 shows the nitrogen gas adsorption and desorption isotherms of the series of TiO_2_ samples; all of these curves can be classified as type IV isotherm characteristic of mesoporous materials with the presence of a hysteresis loop in the relative-pressure range of 0.6–1.0^44^,^45^. The specific surface areas and average pore diameters of the synthesized TiO_2_ were analyzed based on nitrogen adsorption and desorption measurements (Table 1). There is no remarkable change between pristine TiO_2_ and TiO_2_-1, both of which have a BET surface area of around 78.8 m^2^ g^−1^, while TiO_2_-2 shows a remarkable decrease in surface area and only has a BET surface area of 44.6 m^2^ g^−1^. Notably, TiO_2_-3 shows a BET surface area of 87.9 m^2^ g^−1^ that is substantially higher than that of the pristine TiO_2_. However sample TiO_2_-4 again shows a significantly decreased surface area of 49.4 m^2^ g^−1^. The pore size distribution was estimated by employing the BJH (Barret-Joyner-Halenda) method. TiO_2_-4 shows an average pore size of 97.8 Å that is significantly lower than those of other samples. It should be noted that the hierarchical structure could be beneficial for enhancing the surface area of a material, while the particle size and the pore volume are also key factors affecting the surface area. Both SEM images and calculations using Scherrer’s equation based on XRD patterns suggest the TiO_2_-4 sample possesses the largest particle size compared with other samples, which might offset the effect from the hierarchical structure.

**Figure 6:**
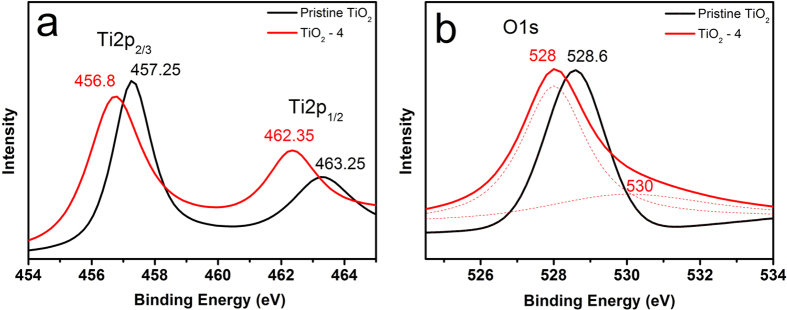
X-ray photoelectron spectra (XPS) of (**a**) Ti2p and (**b**) O1s of pristine TiO_2_ and TiO_2_-4.

X-ray photoelectron spectroscopy (XPS) measurements were carried out to investigate the chemical states and electronic structure of Ti^4+^ in pristine TiO_2_, TiO_2_-3 and TiO_2_-4. As presented in Fig. 6, the XPS signal of Ti 2p was recorded ranging from 454 to 465 eV for the ristine TiO_2_ and TiO_2_-4. The Ti 2p_3/2_ peak shifts from 457.2 eV of pristine TiO_2_ to 456.8 eV for TiO_2_-4 accompanying with the negative shift of Ti 2p_1/2_ peak from 463.2 eV to 462.4 eV, suggesting the partial reduction of TiO_2_ with the formation of Ti^3+^ on the surface of the as-prepared TiO_2_-4. The existence of Ti^3+^ in the sample TiO_2_-4 was also confirmed by the X-band Electron Paramagnetic Resonance (EPR) spectra, as shown in Fig. S5.^46^,^47^,^48^. Based on the EPR results, it is found that TiO_2_-4 shows a peak intensity of ca. 561, which is three times higher than that of the pristine TiO_2_ (160.4). Because the intensity signal of EPR evidences the amount of unpaired electrons, it is reasonable to conclude that the amount of Ti^3+^ ions in the TiO_2_-4 sample is much higher than that in the pristine TiO_2_^49^. Also signals with g values in the range of 2.0 to 2.08 are belong to photogenerated holes that are trapped by the subsurface lattice oxygen. It is generally agreed that the holes are located at oxygen vacancies which react with the O^2−^ and OH^–^ to form 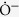 and 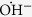 radicals on the surface of catalysts for oxidative decomposition of organic materials. Based on the integrated area of the signals, a larger amount of O^−^ radicals present on the surface of Ti^3+^-doped materials resulted in more effective photocatalysis^49^. It should be noted that the energy difference between XPS Ti2p 3/2 and Ti2p 1/2 peaks for the sample TiO_2_-4 is ca. 5.55 eV; this value is slightly lower than that of the pristine TiO_2_ (ca. 6.0 eV)^50^,^51^,^52^. The slight change in energy difference of the Ti2p peaks can be attributed to the formation of a mixed phase of rutile and anatase in the sample TiO_2_-4^53^. In addition to Ti^3+^, oxygen vacancies can also be possibly produced during the hydrothermal process^54^,^55^. Figure 6b exhibits the O 1s XPS spectra of the pristine TiO_2_ and TiO_2_-4. The Ti-O peak shifts from 528.6 eV for the pristine TiO_2_ to 528 eV for the TiO_2_-4; in addition, a new peak located at 530 eV is attributed to Ti-OH, confirming the formation of hydroxyl group on the TiO_2_ surface after the hydrogen treatment^22^,^56^. We also observed the similar O 1s peak broadening and identical Ti 2p peaks in the as-prepared sample TiO_2_-3 (ESI, Fig. S3).

**Figure 7:**
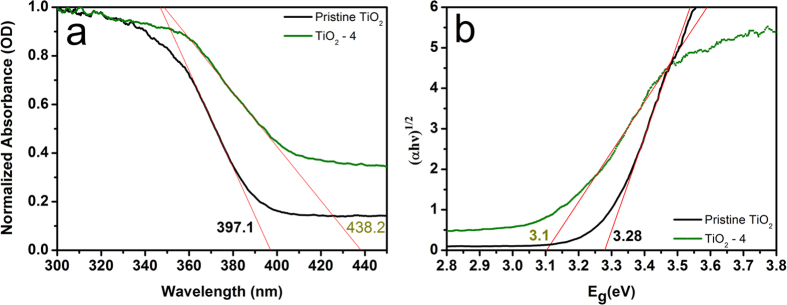
(**a**) UV-visible diffuse reflectance spectra of pristine TiO_2_ and TiO_2_-4. (**b**) Curve -fitting by using the Kubelka-Munk function method for the calculated absorbance against the photon energy for the pristine TiO_2_ and TiO_2_-4.

UV-visible diffuse reflectance spectra were obtained to investigate the light absorption characteristics of the series of TiO_2_ samples (ESI, Fig. S4). The absorption edges are measured to be 397.1 nm, 406.0 nm, 394.7 nm, 411.9 nm and 438.2 nm for pristine TiO_2_, TiO_2_-1, TiO_2_-2, TiO_2_-3 and TiO_2_-4 respectively. As is well known, the positive shift of the absorption spectra of the photocatalyst is in favor of enhancing photocatalytic performance. It should be noted the variation in the intensity of the spectra background could be attributed to the amount of TiO_2_ samples used for testing or the particle size of the samples. Figure 7a shows diffuse reflectance spectra of pristine TiO_2_ and as-prepared TiO_2_-4. It can be seen the absorption onset is around 397.1 nm for pristine TiO_2_, but this absorption extends into the visible region (438.2 nm) for TiO_2_-4, which can be attributed to the Ti^3+^ doping, the crystallite size, and the phase structure of the samples. The red shift of absorption edge indicates a decrease in the band gap. The corresponding band gap energy value was obtained by plotting the Kubelka-Munk function against the photon energy, as shown in Fig. 7b 57,58. The band gap energy value of TiO_2_-4 is 3.1 eV, which is smaller than that of pristine TiO_2_ (3.28 eV).

**Figure 8:**
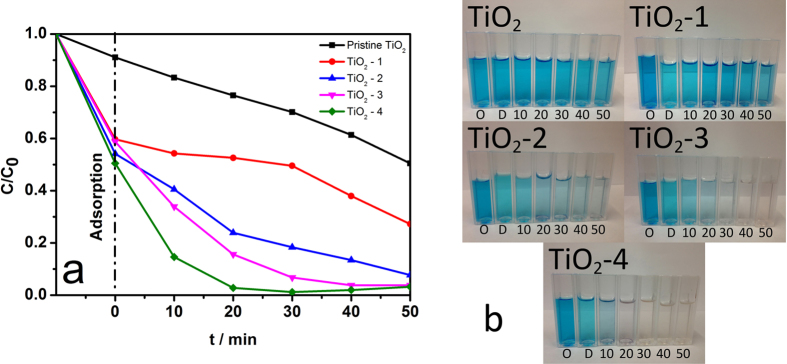
(**a**) Photocatalytic degradation rate of methylene blue vs. irradiation time using pristine TiO_2_ and as-synthesized TiO_2_ samples. (**b**) The evolution of photodegradation of methylene blue solution under visible-light irradiation. (O: Original methylene blue aqueous solution; D: Dark environment; 10~50: 10~50 minutes visible light irradiation)

Photocatalytic reactions for the degradation of methylene blue (MB) aqueous solution were performed to investigate the photocatalytic activity of the series of TiO_2_ samples, as shown in Fig. 8. All of the TiO_2_ samples after the hydrothermal treatment showed an enhanced photodegradation rate for MB compared with the pristine TiO_2_ under simulated sunlight irradiation (AM 1.5 G and 100 mW cm^−2^). The evolution of methylene blue solution, under 10 minutes dark environment and 50 minutes visible light irradiation, are shown in Fig. 8b. Among the samples after hydrothermal reactions, the TiO_2_-4 catalyst showed the highest photocatalytic activity. After irradiation for 20 min, nearly 97.2% of MB was degraded by the sample TiO_2_-4. The TiO_2_-4 sample was far more efficient than any other samples TiO_2_-3, TiO_2_-2, TiO_2_-1, and pristine TiO_2_ that present a degradation percentage of about 84.3%, 76.1%, 47.4%, and 23.5%, respectively. It should be noted that, in the dark environment, the TiO_2_-4, despite of a relatively lower BET surface area, shows a significantly improved adsorption capability compared with pristine TiO_2_, indicating the Ti^3+^ on the surface of TiO_2_-4 may also play a key role in promoting the capability to adsorb the organic dye, thereby leading to an outstanding photocatalytic activity^59^.

## Discussion

Our work has demonstrated an improved approach to realize controllable synthesis of Ti^3+^-doped TiO_2_ by hydrothermal method using sodium borohydride (NaBH_4_) as a reductant. In comparison with the method reported previously, the as-prepared Ti^3+^-doped TiO_2_ could be synthesized using a facile and convenient hydrothermal method. During the hydrothermal process, NaBH_4_ can act as a reductant directly or hydrolyze to release the reductive H_2_ (Reaction 2). In such a reducing atmosphere, the reduction of Ti^4+^ is facilitated by atomic hydrogen with the generation of Ti^3+^ on the TiO_2_ surface (Eq. 3).


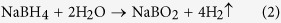



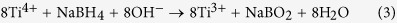



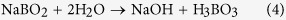


With the increasing amount of NaBH_4_ applied in the hydrothermal treatment, more hydrogen was released from the NaBH_4_ hydrolytic process to generate a higher pressure at a mild temperature. Therefore, the TiO_2_-4 sample could have the highest defect concentration. In addition, the high concentration of NaBH_4_ not only induces a higher pressure due to the generation of H_2_, but also results in stronger alkali environment that originates from further hydrolysis of NaBO_2_ (Reaction 4). Under such a condition, part of anatase TiO_2_ transformed into rutile TiO_2_ with the evolution of nanoparticles into hierarchical structures. According to the XPS results, Ti 2p peaks of TiO_2_ shift to a lower binding energy, confirming the presence of Ti^3+^ decorating on the surface of as-obtained TiO_2_-4. In addition, oxygen vacancies are also produced during the hydrothermal process which can trap photo-excited electrons together with additional formation of Ti^3+^. Thus, it is reasonable that the TiO_2_-4 sample possesses the highest photocatalytic activity since the hierarchical structure can multiply UV light absorption which results in a high efficiency of light-harvesting. Moreover, given the fact that P25 TiO_2_ with mixed phases of rutile and anatase possess a higher catalytic activity than pure phase rutile and anatase TiO_2_ and TiO_2_-4 exhibited the highest photocatalytic degradation efficiency of methylene blue despite the fact that the BET surface area of TiO_2_-4 is smaller than those of the pristine TiO_2_ and TiO_2_-3, it is reasonable to deduce that the hierarchical structure, the mixed phase (rutile and anatase), and the Ti^3+^ defects in the TiO_2_-4 may synergistically contribute to enhancing the catalytic activity. It should be noted that the band gap of TiO_2_-4 based on the Kubelka-Munk function is 3.1 eV, which is slightly smaller than that of pristine TiO_2_ (3.28 eV), confirming that adding NaBH_4_ as a reductant causes the absorption edge of TiO_2_ to shift to a lower energy region. Therefore, this study may offer a simple and low-cost route to functionalize the TiO_2_ and enhance its visible light absorption ability with a narrowed band gap, thereby leading to an improved photocatalytic activity.

In summary, a set of Ti^3+^-doped TiO_2_ samples with controllable photocatalytic properties were designed and prepared using a hydrothermal method *via* tuning the amount of NaBH_4_. The as-developed method showed a well-controlled manner in tuning the surface properties of TiO_2_, as evidenced by color changes from white, light yellow, light grey, to dark grey upon adjusting the amount of NaBH_4_. In addition, we firstly reported that, with a high concentration of NaBH_4_ applied in the hydrothermal reaction, a high pressure and strong alkali environment were introduced to facilitate the conversion of anatase TiO_2_ into rutile TiO_2_ with the evolution of nanoparticles into hierarchical structures. More importantly, it is demonstrated that the as-developed Ti^3+^-doped TiO_2_ with a mixed phase and nanostructure can potentially lower the recombination rate of electron-hole pairs due to the presence of Ti^3+^ and oxygen vacancies that are able to trap photo-excited electrons on the surface. Furthermore, with the absorption edge of TiO_2_ shifting to the visible-light region by adding NaBH_4_ as a reductant, the synthesized TiO_2_ is expected to exhibit a higher photocatalytic activity and efficiency.

## Methods

### Preparation of Ti^3+^-doped titanium dioxide

To fabricate the Ti^3+^-doped TiO_2_, a two-step hydrothermal synthesis procedure was implemented. First, 5 ml of 50 wt. % titanium (IV) bis (ammonium lactato) dihydroxide (purchased from Sigma-Aldrich) solution was dispersed in 60 ml 0.08 g/L glucose with stirring for 0.5 hour. 65 ml of the above solution was then transferred into an autoclave for hydrothermal reactions at 170 ∞C for 8 hours. Then the products were washed by deionized water and ethanol for 4 times each and filtered. After the calcination treatment at 500 ∞C for 3 hours, dried TiO_2_ powders were obtained. Different amounts of sodium borohydride (purchased from Alfa Aesar) caplets were directly added into 60 ml water and mixed with 0.50 g TiO_2_ powder for hydrothermal reactions in an autoclave at 180 ∞C for 16 hours. Finally, the Ti^3+^-doped titanium dioxide powders were collected by filtration, washed alternately 3 times with deionized water and ethanol, and then dried at 60 ∞C in air for 10 hours.

### Material Characterizations

The X-ray powder diffraction (XRD) analyses were conducted on a Scintag XDS 2000 diffractometer equipped with a scintillation counter and Cu k-alpha radiation (0.154056 nm) reflection mode. The microscopic morphology and structures of the samples are obtained using a Hitachi (S-4800) scanning electron microscope (SEM) and Hitachi H-9000NAR transmission electron microscope (TEM). X-ray photoelectron spectroscopy (XPS) was conducted by using VG ESCA 2000 with an Mg Kα as source and the C1s peak at 284.5 eV as an internal standard. The specific surface area was obtained using ASAP2020 (Micromeritics, U.S.A) Brunauer-Emmett-Teller (BET) nitrogen adsorption-desorption. The N_2_ adsorption-desorption measurements were carried out at 77 K using a Quantachrome Autosorb gas-sorption system. The samples were degassed at 180 ∞C for 2 hours before the measurements. The Raman spectra of the Ti^3+^-doped TiO_2_ powders were measured using a Raman microscope (Brucker RFS 100/S spectrometer) with an excitation wavelength of 1,064 nm at an input power of 1 mW. The optical absorption spectroscopy measurements were obtained using an Ocean Optics SD2000 UV-visible spectra spectrometer with a closed quartz cell (optical path length: 1 cm).

### Photocatalytic reaction

30 mg of the powder samples were ultrasonically dispersed in 50 mL deionized water followed by the addition of 0.01 g / L methylene blue (MB) aqueous solution. The mixture was then stirred under darkness for 10 minutes to achieve adsorption-desorption equilibrium. Subsequently, the suspension with continuous stirring was exposed under a Xe lamp (AM 1.5 G and 100 mW cm^−2^) with an incident direction normal to the surface of the solution. At given irradiation intervals, 3 mL aliquots of the suspension were collected and separated by centrifugation. The absorption spectrum of the supernatant was measured using a UV-Vis spectrometer (Ocean Optics SD2000). The concentration of MB was determined by monitoring the changes in the absorbance maximum at 662.6 nm.

## Additional Information

**How to cite this article**: Ren, R. *et al.* Controllable Synthesis and Tunable Photocatalytic Properties of Ti^3+^-doped TiO_2_. *Sci. Rep.* **5**, 10714; doi: 10.1038/srep10714 (2015).

## Supplementary Material

Supplementary Information

## Figures and Tables

**Table 1:** Surface properties of pristine TiO_2_ and as-synthesized TiO_2_.

**Sample**	**BET Surface Area (m^2^/g)**	**Adsorption average pore width (Å)**	**Total pore volume (cm^3^/g)**
**Pristine TiO**_**2**_	78.9	171.8	0.339
**TiO**_**2**_**– 1**	78.8	197.5	0.389
**TiO**_**2**_**– 2**	44.6	171.2	0.191
**TiO**_**2**_**– 3**	87.9	145.2	0.319
**TiO**_**2**_**– 4**	49.4	97.8	0.121
