# Supplementary material for: Controllable Synthesis and Tunable Photocatalytic Properties of Ti3+-doped TiO2
Source: Sci Rep. 2015 Jun 5;5:10714. doi: 10.1038/srep10714 (PMC4650606; doi:10.1038/srep10714)
Supplement: Supplementary Information [file srep10714-s1.pdf]

Supplementary Information

Controllable Synthesis and Tunable Photocatalytic Properties of Ti^{3+}-doped TiO_2

Ren Ren, Zhenhai Wen*, Shumao Cui, Yang Hou, Xiaoru Guo, Junhong Chen*

Department of Mechanical Engineering, University of Wisconsin-Milwaukee, 3200 North Cramer Street, Milwaukee, WI 53211, USA

E-mail: wenzhenhai@yahoo.com; *jhchen@uwm.edu*

This document contains the following supplementary information:

1. SEM images of the as-synthesized TiO_2 nanostructures
2. TEM micrographs of sample TiO_2-3
3. UV-visible diffuse reflectance spectra of pristine TiO_2 and synthesized TiO_2
4. X-ray photoelectron spectra (XPS) of pristine TiO_2 and synthesized TiO_2
5. EPR spectra of the pristine TiO_2 and TiO_2-4 at 100 K under N_2 atmosphere.

1. SEM images of the as-synthesized TiO_2 nanostructures

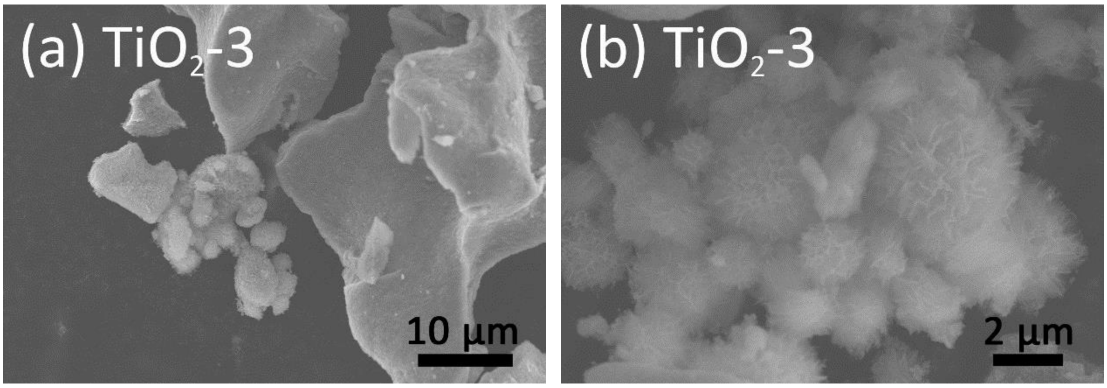

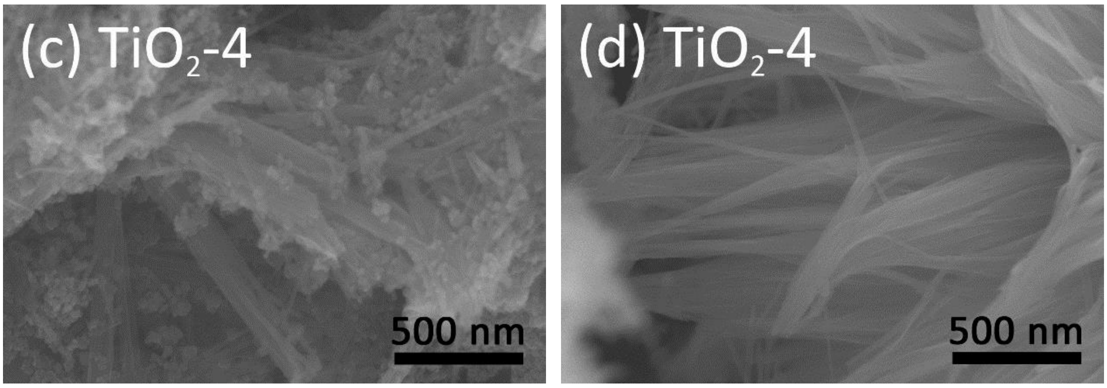

Fig. S1. SEM images of as-synthesized TiO_2-3 (a) and TiO_2-4 nanostructures (b-d)

2. TEM micrographs of sample TiO_2-3

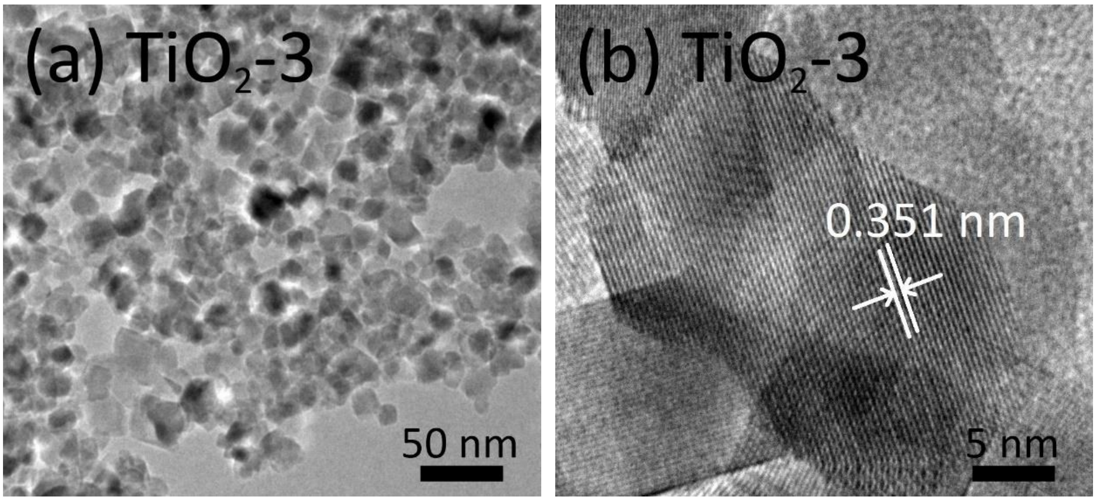

Fig. S2. TEM micrographs of sample TiO_2-3: (a) overview image of TiO_2-3; (b) HRTEM image of TiO_2-3.

3. UV-visible diffuse reflectance spectra of pristine TiO_2 and synthesized TiO_2

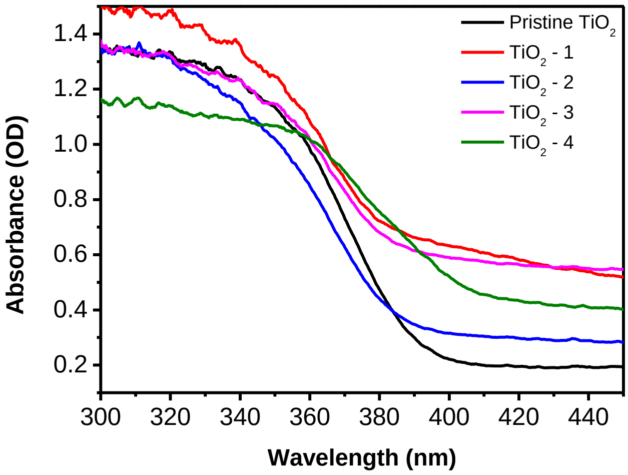

Fig. S3. UV-visible diffuse reflectance spectra of pristine TiO_2 and synthesized TiO_2.

4. X-ray photoelectron spectra (XPS) of pristine TiO_2 and synthesized TiO_2

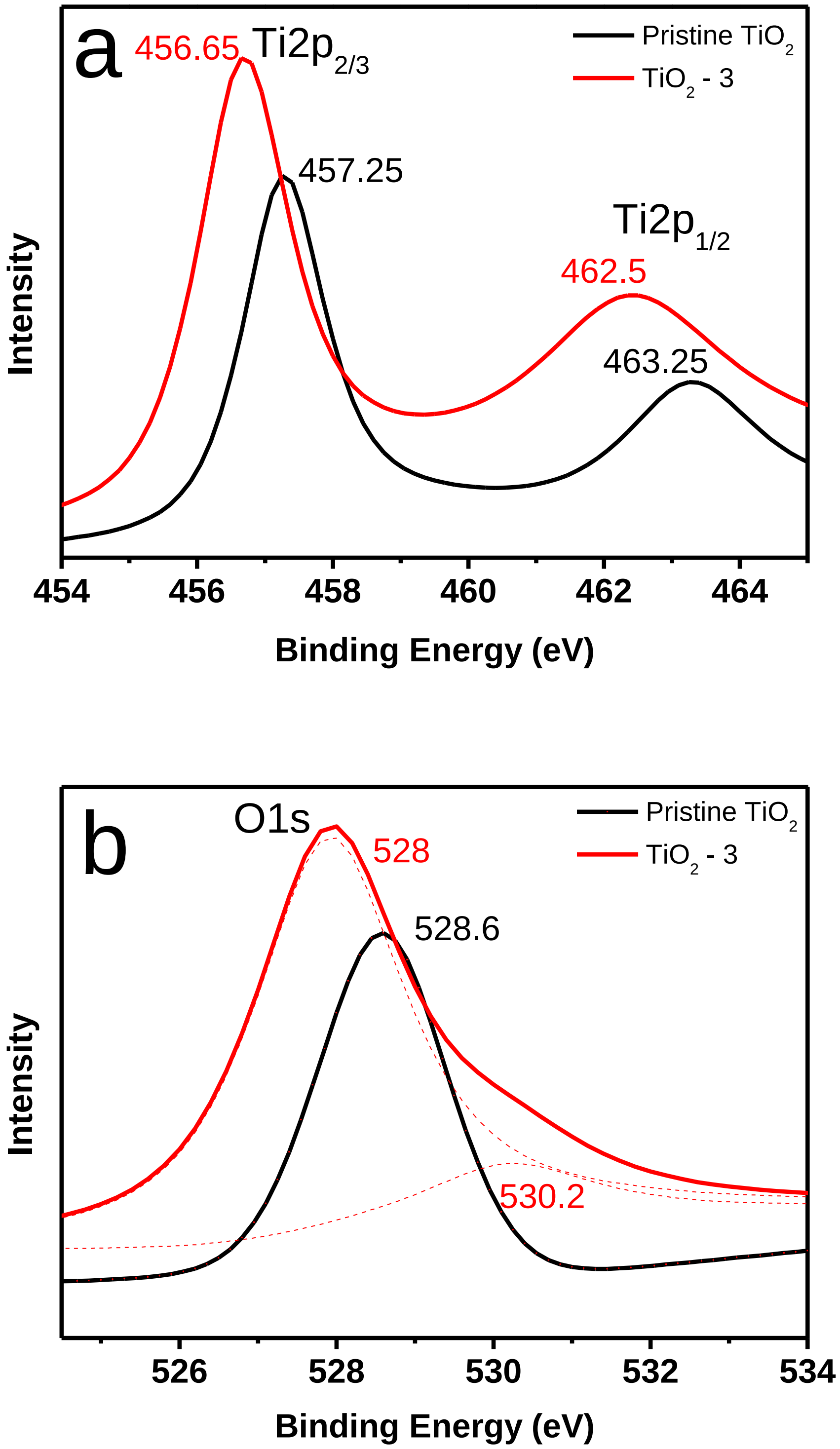

Fig. S4. X-ray photoelectron spectra (XPS) of (a) Ti2p and (b) O1s of pristine TiO_2 and TiO_2-3.

5. EPR spectra of the pristine TiO_2 and TiO_2-4 at 100 K under N_2 atmosphere.

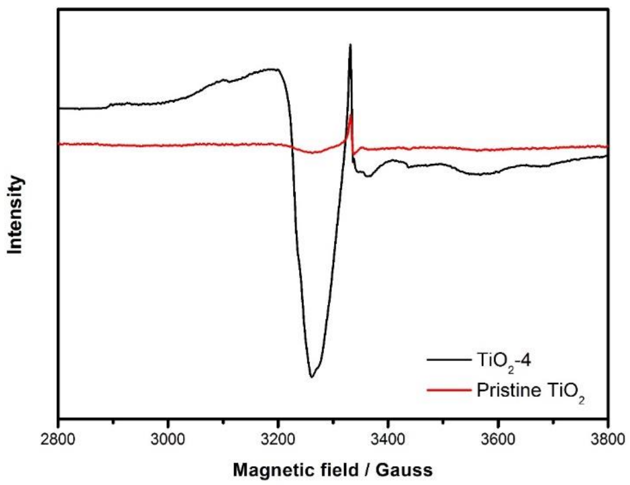

Fig. S5. EPR spectra of the pristine TiO_2 and TiO_2-4 at 100 K under N_2 atmosphere.
